# Habitat filtering shapes the differential structure of microbial communities in the Xilingol grassland

**DOI:** 10.1038/s41598-019-55940-y

**Published:** 2019-12-18

**Authors:** Jie Yang, Yanfen Wang, Xiaoyong Cui, Kai Xue, Yiming Zhang, Zhisheng Yu

**Affiliations:** 1 College of Resources and Environment, University of Chinese Academy of Sciences, Beijing, 100049 China; 2 College of Life Sciences, University of Chinese Academy of Sciences, Beijing, 100049 China; 3 Beijing Municipal Ecological Environment Bureau, Beijing, 100048 China; 4 Research Center for Eco-Environmental Sciences, Chinese Academy of Sciences, Beijing, 100085 China

**Keywords:** Grassland ecology, Microbial ecology

## Abstract

The spatial variability of microorganisms in grasslands can provide important insights regarding the biogeographic patterns of microbial communities. However, information regarding the degree of overlap and partitions of microbial communities across different habitats in grasslands is limited. This study investigated the microbial communities in three distinct habitats from Xilingol steppe grassland, i.e. animal excrement, phyllosphere, and soil samples, by Illumina MiSeq sequencing. All microbial community structures, i.e. for bacteria, archaea, and fungi, were significantly distinguished according to habitat. A high number of unique microorganisms but few coexisting microorganisms were detected, suggesting that the structure of microbial communities was mainly regulated by species selection and niche differentiation. However, the sequences of those limited coexisting microorganisms among the three different habitats accounted for over 60% of the total sequences, indicating their ability to adapt to variable environments. In addition, the biotic interactions among microorganisms based on a co-occurrence network analysis highlighted the importance of *Microvirga*, *Blastococcus*, *RB41*, *Nitrospira*, and four norank members of bacteria in connecting the different microbiomes. Collectively, the microbial communities in the Xilingol steppe grassland presented strong habitat preferences with a certain degree of dispersal and colonization potential to new habitats along the animal excrement- phyllosphere-soil gradient. This study provides the first detailed comparison of microbial communities in different habitats in a single grassland, and offers new insights into the biogeographic patterns of the microbial assemblages in grasslands.

## Introduction

A central goal in both microbial ecology and biogeography is to explore the variation in microbial abundance and diversity in different ecosystems^1,2^. In comparison to aquatic systems, soils are particularly heterogeneous, and numerous environmental factors are assumed to control the spatial patterns of microorganisms^3,4^. Grasslands, as a major components of the global ecosystem, cover 37% of the terrestrial area on Earth^5^. With an extraordinarily high diversity of plants and herbivores, grasslands provided a well-structured platform for exploring the interaction of a range of biotic and abiotic factors that alter the structure of soil microbial communities^6–8^. Illuminating the spatial patterns of microorganisms in a grassland ecosystem not only helps to predict the critical processes that occur within grasslands (e.g. grassland degradation and restoration) but also helps to build a more comprehensive microbial biogeographic pattern at a larger scale^9,10^.

In grasslands, aboveground and belowground communities are intrinsically linked and the feedbacks between these subsystems have important implications for biogeochemical cycles and grassland ecosystem functioning^11–13^. The large number of microorganisms that inhabit the aboveground and belowground niches in grasslands play vital roles in nutrient cycling and contribute greatly to grassland health^14,15^. Previous research suggests that plant growth-promoting microorganisms (PGPM), which commonly reside in the phyllosphere and rhizosphere, can exhibit beneficial effects on plant growth by facilitating nitrogen, phosphorus, and iron uptake from the soil via nitrogen fixation, inorganic phosphate solubilisation, and the production of iron chelators, respectively^16^. In addition, 30–50% of aboveground plant biomass is consumed annually by large herbivores^17,18^, and the decomposition of animal excrement is a significant pathway for nutrient cycling through the mutual effect of faecal and soil microorganisms^19,20^. Thus, in grassland ecosystems, microorganisms inhabited in animal excrement, plants, and soil constitute a complex distribution pattern which required in-depth investigation and understanding.

In fact, mountainous studies have focused on the microbial assemblages among different habitats in a range of systems including forests^21^, rock glacier-ponds^22^, peatland systems^23^, terrestrial- freshwater gradients^24^, and the human body^25^. Although the crossing of boundaries between differential habitats enables microorganisms to occupy novel niches and explore accessible nutrient resources, it is a large challenge for microorganisms^26^. Both deterministic (e.g. environmental conditions and biotic interactions) and stochastic processes (e.g. dispersal limitation, the mass effect, and random events) have been used to explain microbial community assemblages^27–30^. Previous studies have shown that different mechanisms occur simultaneously and are co-responsible for the structure of microbial assemblages^31,32^. For example, different ecological groups, such as habitat generalists and specialists, exhibit different organismal traits and exhibit different responses to varying environmental conditions^33^. Therefore, the microbial assemblages for habitat generalists and specialists may vary according to their tolerance to different environmental conditions^34^. In addition, habitat heterogeneity and connectivity also affect microbial assemblages on a local scale^23,35^. As a typical representative of a multicomponent terrestrial ecosystem, grasslands show complex interactions across the animal-grass-soil gradient^36–38^. However, compared with other ecosystems, information regarding the microbial assemblages across the distinct habitats of animal excrement-phyllosphere-soil in grasslands is still limited. Whether or not these different habitats have significant differential microbial community structure? What is the degree of overlap in microbial communities among the three habitats? And what are the keystone microorganisms in connecting among microbiomes?

In the present study, we investigated the microbial communities in three different habitats in a grassland ecosystem, i.e. animal excrement, phyllosphere, and soil. The difference and similarity of microbial community structure among different habitats in grassland ecosystem was analysed using Illumina sequencing of amplicon libraries that targeted bacteria, archaea, and fungi. In addition, the keystone microorganisms in connecting among microbiomes in this animal excrement-plant-soil system were also identified. This study serves to advance our knowledge of the microbial ecology of different grassland habitats and to improve analysis regarding the impact of microbial communities on the health and functioning of grasslands.

## Results

### Microbial diversity among different habitats in grassland

In total, 590,663 bacterial 16 S rRNA gene sequences were obtained from 12 samples from the 3 grassland habitats, which were then clustered into 4,382 operational taxonomic units (OTUs). Furthermore, 586,063 archaeal sequences (164 OTUs) and 638,002 ITS sequences (1,766 OTUs) were retrieved from 16 S rRNA and ITS gene sequencing, respectively. To compare the samples with the same sequencing depth, normalization of the sequence number was conducted by extracting the minimum number of bacterial (17,247), archaeal (36,770), and fungal (42,275) sequences in each sample (Supplementary Figs. S1–S3).

The richness of microbial communities in the soil was significantly greater than that in the animal excrement for all three microbial groups (Duncan test, P < 0.05), but the microbial diversity in the phyllosphere varied among the samples, with a higher similarity to the fungal communities in the soil as well as to the bacterial and archaeal communities in the animal excrement. The trends of the Chao 1 estimator and Shannon index in all three habitats were all consistent with the results of OTU richness, except for the Shannon index in the bacterial communities (Table 1).

**Table 1:** Comparison of alpha diversity indexes among the three habitats.

Habitat	Richness	Chao1	Shannon	Good’s coverage
**Bacteria**				
Animal excrement	625 ± 215^b^	813 ± 442^b^	5.04 ± 0.13^a^	0.9912 ± 0.009^a^
Phyllosphere	1082 ± 625^b^	1517 ± 805^b^	4.45 ± 2.33^a^	0.9778 ± 0.0126^b^
Soil	1605 ± 212^a^	2122 ± 287^a^	6.10 ± 0.27^a^	0.9707 ± 0.0043^b^
**Archaea**				
Animal excrement	30 ± 8^b^	30 ± 8^b^	1.76 ± 0.56^b^	0.9999 ± 0^a^
Phyllosphere	58 ± 16^a^	61 ± 15^a^	2.49 ± 0.44^a^	0.9999 ± 0^a^
Soil	63 ± 8^a^	67 ± 12^a^	2.66 ± 0.18^a^	0.9998 ± 0.0001^a^
**Fungi**				
Animal excrement	274 ± 51^b^	340 ± 63^b^	2.24 ± 0.16^b^	0.9982 ± 0.0003^a^
Phyllosphere	279 ± 60^b^	409 ± 76^b^	2.44 ± 0.29^b^	0.9978 ± 0.0006^a^
Soil	515 ± 97^a^	616 ± 139^a^	3.71 ± 0.48^a^	0.9973 ± 0.0009^a^

Richness, observed number of OTUs; Chao 1, Chao 1 richness estimator; Shannon, Shannon diversity index; Good’s coverage, Good’s coverage estimates. Values represent means ± S.D. Values with different letters in the same columns indicate significant differences among the three habitats (Duncan’s test, P < 0.05).

### Microbial composition among the different grassland habitats

#### Comparison of the bacterial compositions among the three habitats

**Figure 1:**
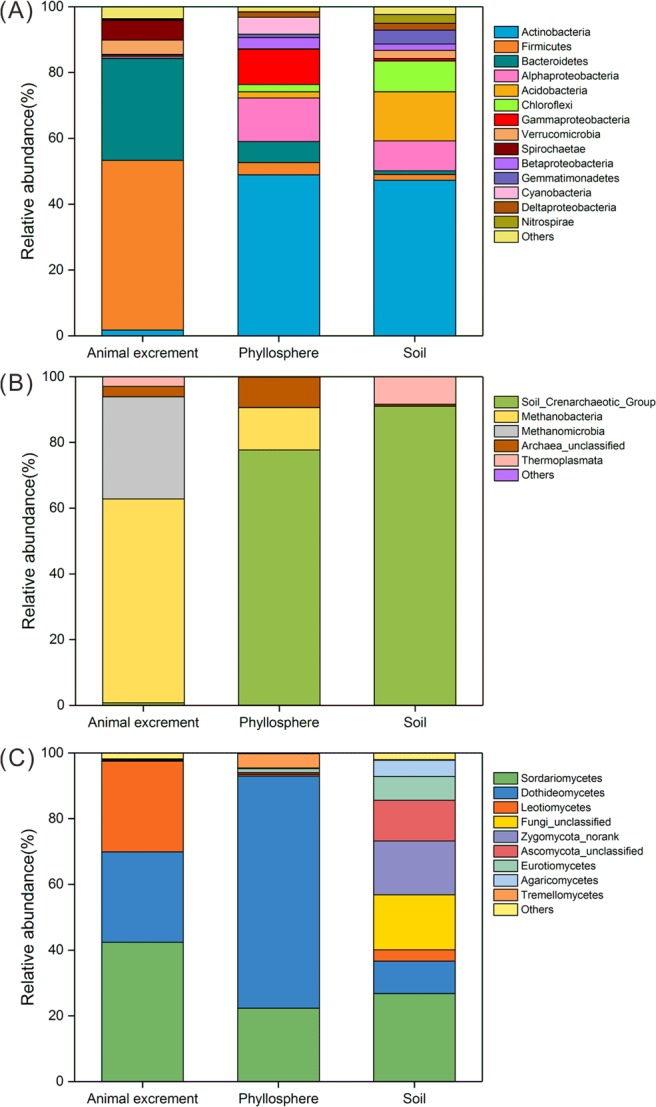
Taxonomic information of animal excrement, phyllosphere, and soil samples for bacteria (**A**), archaea (**B**), and fungi. (**C**) Bacterial communities were visualized at the phylum level, while archaeal and fungal communities were visualized at the class level. The phyla or classes with an average abundance of less than 1% were classified as “others”.

At the phylum level, Firmicutes and Bacteroidetes accounted for >80% of the bacterial communities in the animal excrement. Other bacterial phyla, including Spirochaetae, Verrucomicrobia, Fibrobacteres, and Actinobacteria, were also found to be minor groups (>1%) in this habitat. In the phyllosphere samples, Actinobacteria was the most abundant phylum, accounting for nearly half of the bacterial sequences in the phyllosphere samples. In addition, nearly 47% of the bacterial sequences of the soil samples (i.e. 42% from shallow soil samples and 53% from deep soil samples, on average) were derived from phyla Actinobacteria. Acidobacteria (15%) and Alphaproteobacteria (9%) also comprised the major bacterial components in the soil samples, and they were found in relatively high abundance in shallow soil samples (Fig. 1A).

#### Comparison of the archaeal compositions among the three habitats

In the animal excrement samples, the archaeal communities were mainly comprised of phylum Euryarchaeota, which accounted for 96% of the archaeal sequences, followed by unclassified archaea (3%) and Thaumarchaeota (1%). Specifically, classes Methanobacteria and Methanomicrobia were the dominant archaea with 62% and 31% of the archaeal sequences in excrement samples, respectively. Phyla Thaumarchaeota and Euryarchaeota accounted for > 90% of the archaeal sequences from the phyllosphere samples, and Soil Crenarchaeotic Group (78%) was the most dominant class in the phyllosphere, followed by Methanobacteria (13%) and unclassified archaea (9%). In the soil samples, Thaumarchaeota accounted for >90% of the archaeal sequence data, and the Soil Crenarchaeotic Group was the only class in this phylum (Fig. 1B).

#### Comparison of the fungal compositions among the three habitats

In the fungal communities, the phylum Ascomycota was clearly dominant in all samples, especially accounting for 99% in the animal excrement samples. Furthermore, classes Sordariomycetes (42%), Leotiomycetes (28%), and Dothideomycetes (27%), belonging to phylum Ascomycota, were found in high abundance in the animal excrement samples. In the phyllosphere samples, class Dothideomycetes was predominant (71%), followed by classes Sordariomycetes and Tremellomycetes (accounting for 22% and 4% of the fungal sequences, respectively). In the soil samples, Ascomycota accounted for 60% of the fungal sequences and was mainly composed of classes Sordariomycetes (27%) and Dothideomycetes (10%), and unclassified members of Ascomycota (12%) (Fig. 1C).

#### Differentiation of the microbial communities across the different habitats

**Figure 2:**
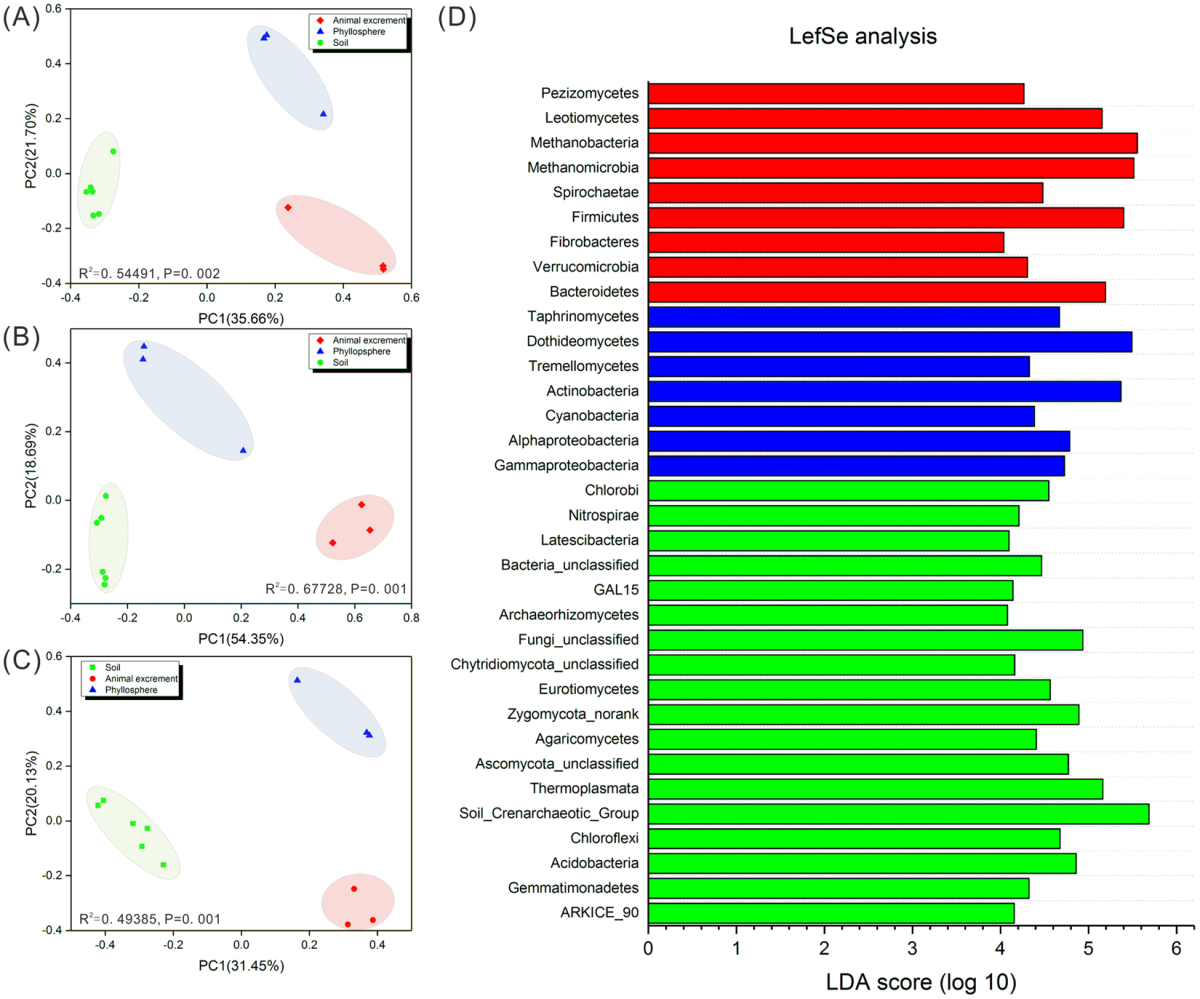
Principal coordinates analysis (PCoA) of the microbial communities for bacteria, archaea, and fungi (**A**–**C**) and linear discriminant analysis of the effect size (LefSe) of microorganisms in different habitats. Variation between animal excrement, phyllosphere, and soil samples were calculated based on the Bray-Curtis distance matrix. Similarity values based on a permutational multivariate analysis of variance (PERMANOVA) are shown in plots. (**A**–**C**) A linear discriminant analysis threshold score of 4.0 and a significant threshold score of 0.05 were set for the LefSe analysis. The levels of classification in the LefSe analysis are the same as those used in Fig. 1.

Microbial comparison analysis were performed to determine the changes in microbial composition among three different habitats, i.e. animal excrement, phyllosphere, and soil. The principal coordinates analysis (PCoA) plot, based on Bray-Curtis distances, revealed the differentiation of the composition of the microbial communities across the three habitats (Fig. 2A–C). Samples in all three microbial groups were clustered by habitat, with a significant separation among animal excrement, phyllosphere, and soil samples (PERMANOVA, P < 0.005). Similar results were shown in the hierarchical clustering dendrogram (Supplementary Fig. S4). Microbial communities were differentiated among the different habitats, except for a weak differentiation in the bacterial communities between animal excrement and phyllosphere samples.

To determine the microorganisms that most likely explained the observed differences among samples from the three habitats, we performed a linear discriminant analysis of the effect size (LEfSe). The LEfSe analysis revealed the representative microorganisms in the three habitats, including 9 taxa in the animal excrement samples, 7 taxa in the phyllopshere samples, and 18 taxa in the soil samples (Fig. 2D). Animal excrement samples were enriched with bacteria, such as Firmicutes, Bacteroidetes, Spirochaetae, Verrucomicrobia, and Fibrobacteres; archaea, such as Methanobacteria and Methanomicrobia; and fungi, such as Leotiomycetes and Pezizomycetes. Phyllosphere samples were enriches with bacteria, such as Actinobacteria, Alphaproteobacteria, Gammaproteobacteria, and Cyanobacteria; and fungi, such as Dothideomycetes, Taphrinomycetes, and Tremellomycetes. Soil samples were enriched with bacteria, such as Acidobacteria and Chloroflexi; archaea, such as Soil Crenarchaeotic Group and Thermoplasmata, and fungi, such as Eurotiomycetes and Agaricomycetes.

#### Habitat overlap and partitioning between microbial communities

**Figure 3:**
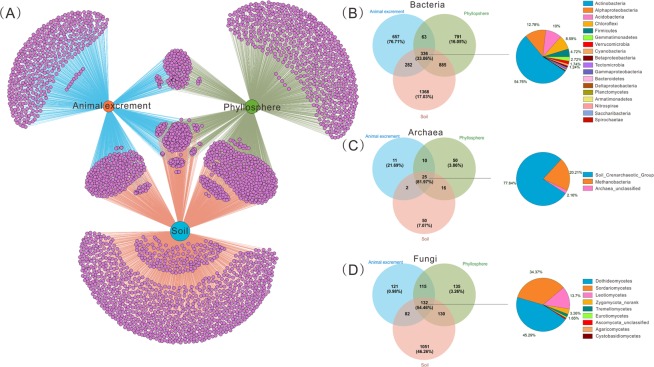
Operational taxonomic unit (OTU) network map (**A**) and Venn diagrams of shared and unique OTUs in three grassland habitats. (**B**–**D**) The proportions of sequences associated with the unique or shared OTUs in the total sequences obtained from each or all three habitats are shown in parentheses. The taxonomic data of shared OTUs in the three habitats are presented as the percent contribution of sequences in each microbial phylum or class relative to the total sequences. The levels of classification in the shared OTUs are the same as those used in Fig. 1.

The network and Venn diagrams revealed the overlap and partitioning of the microbial OTUs across three habitats (Fig. 3). Almost 67.08% OTUs were identified as unique OTUs, i.e. only detected in one habitat. Soil samples possessed the highest unique OTUs among the three habitats and over half of the total OTUs obtained from the soil samples were identified as unique OTUs. In the animal excrement and phyllosphere samples, the unique OTUs accounted for 43% and 36% of the total OTUs, respectively. Although these unique OTUs accounted for a high proportion of the total, they generally had a limited relative abundance (Fig. 3B–D), indicating their disadvantageous status in microbial community.

Although relatively few microbial OTUs (493 OTUs, 7.81% of the total OTUs) were shared among the three habitats (Fig. 3A), these coexisting OTUs accounted for 61.13% of the total microbial sequences (Supplementary Table S1). Further analysis revealed that these coexisting OTUs mainly belonged to bacteria Actinobacteria and Alphaproteobacteria, archaea Soil Crenarchaeotic Group, and fungi Dothideomycetes and Sordariomycetes (Fig. 3B–D). The phyllosphere and soil samples shared more OTUs (1524, 27.59% of the total OTUs obtained from both habitats) than coexisting OTUs between animal excrement and phyllosphere (681, 17.72%) or coexisting OTUs between animal excrement and soil (859, 16.10%) (Fig. 3A). It is noteworthy that the pairwise OTUs that coexisted in the phyllosphere and soil were found in higher abundance in the phyllosphere samples (90.02%) than in the soil samples (68.08%).

### Co-occurrence patterns of microbial communities

**Figure 4:**
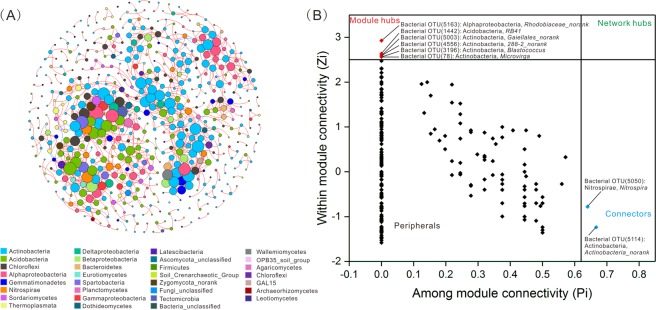
Co-occurrence pattern (**A**) and *Zi*-*Pi* plot (**B**) based on the topological roles of microorganisms. Nodes indicate taxonomic affiliations at operational taxonomic unit (OTU) level. Red and blue lines in the co-occurrence network indicate positive and negative correlations, respectively. The size of each node is proportional to the number of degrees. The classification levels of microorganisms in the co-occurrence network are the same as those used in Fig. 1. The threshold values of *Zi* and *Pi* for categorizing OTUs were 2.5 and 0.62, respectively^80^.

The microbial co-occurrence patterns were explored using a network analysis (Fig. 4A). The microbial network was composed of 556 nodes (OTUs) and 1240 edges, with an average node connectivity degree of 4.46. Among the total microorganisms, the bacterial OTUs were the most representative within the network with 495 nodes, whereas the archaeal and fungal OTUs were represented by 18 and 43 nodes, respectively. The highest degrees of association were detected in a bacterial OTU belonging to Alphaproteobacteria (degree = 20), followed by the OTUs belonging to Acidobacteria, Spartobacteria, and Alphaproteobacteria (degree = 16, Fig. 4A). Within the fungal and archaeal communities, a single fungal OTU belonging to Wallemiomycetes and two archaeal OTUs belonging to Thermoplasmata had the highest degrees of association (13 and 7, respectively). Collectively, Actinobacteria, Acidobacteria, Alphaproteobacteria, and Chloroflexi were widely distributed in this network, accounting for 70% of all nodes (Supplementary Fig. S5). After modularizing the nodes in the network, top three modules were visualized in Supplementary Fig. S6. The nodes from module I mostly belonged to Acidobacteria, Actinobacteria, and Chloroflexi; the nodes from module II mostly belonged to Actinobacteria and Alphaproteobacteria; and the nodes from module III mostly belonged to Actinobacteria and Chloroflexi.

Moreover, the different roles of the nodes in the networks between the three different habitats were identified based on *Zi*-*Pi* plots (Fig. 4B). Most of the OTUs were “peripherals”, suggesting that they had only a few links; most of which were inside their modules. A total of 6 nodes were classified as “module hubs”, indicating that these OTUs were highly connected to many OTUs within their own module. Two nodes were classified as “connectors” which were identified as having among-module connecting roles in the network. Based on the *Pi* and *Zi* value, the eight microorganisms identified as keystone taxa were *Microvirga*, *Blastococcus*, *RB41*, *Nitrospira*, three norank members of Actinobacteria, and one norank member of Alphaproteobacteria.

## Discussion

### Differential microbial diversity and community structures among the three habitats

The Xilingol grassland occurs in the typical steppes of Inner Mongolia, and has a characteristically arid and semiarid environment^39,40^. In the present study, we showed that the soil microbiome harboured a higher diversity of bacterial and fungal communities compared with animal excrement or the phyllosphere (Table 1). In general, the rarefaction curves of the soil bacteria and fungi did not reach saturation at 3% dissimilarity (Supplementary Figs. S1 & S3); therefore, the richness of soil is likely greater than that described in the present study. Although the soil had the highest microbial diversity among the three habitats, the diversity of the bacterial and fungal communities did not differ significantly between the phyllosphere and animal excrement (Table 1). These findings might be explained by the high variance in microbial diversity among the different plant species or animal faeces^41,42^. Meanwhile, insufficient sampling may also account for the weak difference in microbial diversity observed between them. Overall, extensive sampling and a more comprehensive dataset is required to identify the determinants of microbial diversity among different habitats.

The microbial community structures of the different habitats were observed to be differential (Fig. 1). In soil, Actinobacteria, Acidobacteria, and Alphaproteobacteria accounted for 72% of the total bacterial sequences. These findings are consistent with those of Wang *et al*.^43^, who found that these phyla were the most dominant bacteria in typical steppe soil. The majority of archaeal sequences reads belonged to Thaumarchaeota (91.05%), more specifically the ammonia-oxidizing genus *Candidatus Nitrososphaera* and other unclassified members of the Soil Crenarchaeotic Group. Our results are fairly consistent with the information available on archaeal communities from a global soil investigation, i.e. a general dominance of Crenarchaeota (recently classified as Thaumarchaeota), along with minor fractions of Euryarchaeota^44^. Besides, members of the Soil Crenarchaeotic Group were also found to be dominant in savanna soils in South Africa^45^. However, in contrast, a large proportion of the taxa abundant in animal excrement or phyllosphere samples were rare in soil samples and vice versa (Supplementary Fig. S7). For example, members of the bacterial phyla Firmicutes and Bacteroidetes are capable of utilizing starches, cellulose, or non-cellulosic polysaccharides as their primary energy sources, which accounts for their dominance in the gut environment^46^. Digestion of plant biomass in the gut of herbivores also results in the production of methane in the process of carbohydrate fermentation, which accounts for the proliferation of methanogenic archaea^47^. In addition, Dothideomycetes was dominant in all phyllosphere samples in this study (i.e. with a relative abundance of 68%–73%) and, based on a meta-analysis in a global scale^48^, is reportedly the most abundant class in phyllosphere fungal communities. Moreover, Capnodiales, one of the major orders within class Dothideomycetes in the phyllosphere samples in the present study, reportedly have a high tolerance for stressful conditions, including high solar irradiation, extreme temperature shifts, and prolonged desiccation^49^; conditions that are typically characteristic of the phyllosphere environment^42,50^. Overall, the distinct distribution of these microorganisms among the different habitats indicated a significant degree of habitat preference of these microorganisms.

### High number of unique OTUs in different habitats indicated niche differentiation

The Xilingol grassland is a typical temperate grassland that supports a diverse array of herbivores and herbaceous plants. Annually, the herbivores consume the diverse plant species to sustain growth and provide a continuous input of faecal microbiota into the soil microbial pool, which usually enhances the overlap of these habitats. However, the species coexistence theory provides more support for niche differentiation, with coexisting species in the grassland occupying different niches, so as to minimize the competition for limited resources and strengthen the division of labour^51,52^. In the present study, a high number of the microbial taxa were found to be habitat specialists (4234 OTUs, 67.08%), with soil having the highest unique OTU richness (Fig. 3). Especially for fungal communities, the number of unique soil OTUs accounted for more than 75% of the total fungal OTUs observed in soil samples, which indicated that these fungal microorganisms show strong habitat preferences. It is noteworthy that classes Sordariomycetes, Agaricomycetes, and Eurotiomycetes accounted for more than 50% of the unique OTUs in the soil samples when excluded the OTUs of “unclassified fungi” in the present study, and these fungi have previously been reported as common fungi in grassland soil^53,54^. In addition, we found that the bacterial phyla Firmicutes and Bacteroidetes accounted for the majority of the unique microorganisms in animal excrement. These findings are consistent with those of Donnell *et al*.^55^, who identified these two phyla as the core phyla in the faecal microbiota of domesticated herbivorous animals. Meanwhile, more than half the number of unclassified archaea were detected only in the phyllosphere samples, which suggests that an increase in cultivation efforts and more in-depth functional metagenomic explorations are required to describe these unclassified archaea in the phyllosphere environment.

Although a large number of unique OTUs were observed in the three habitats (67.08% of the total number of OTUs), they only accounted for 20.09% of the total sequences. This is consistent with the finding in previous studies, which showed that habitat specialists have lower population densities due to limited efficiencies in finding suitable habitats during the dispersal process^34,56^. Due to the narrow environmental tolerances, habitat specialists are much more vulnerable under fluctuating environmental conditions and are, therefore, more liable to become extinct compared to habitat generalists^57^. In the phyllosphere samples in this study, 36.31% of the OTUs were identified as unique microorganisms, however, these OTUs only comprised 5.78% of the sequences. As previously mentioned, the phyllosphere is a hostile environment for microorganisms, therefore, it is reasonable to infer that the unique microbes in the phyllosphere are undergoing harsh environmental filtering.

### A high abundance of coexisting microorganisms indicates their superior adaption ability

Although only 7.81% of microbial OTUs were shared between all three habitats, these microbes accounted for over 60% of the total sequences. The predominance of these microorganisms might be explained by their high efficiency in finding suitable habitats and survival potential during dispersal, i.e. species with higher competition and adaption abilities tend to be more abundant^34^. Members of Actinobacteria, Proteobacteria, Thaumarchaeota, Dothideomycetes, and Sordariomycetes were detected among three distinct habitats in this study (Fig. 3); which is consistent with their known ubiquitous distributions in the environment^58–62^. Lower resources needs may present a prominent advantage for the ubiquity and proliferation of microbes because they are more resistant to changes in nutrient resources. Bacteria belonging to Acidobacteria, Nitrospirae, Planctomycetes, and Chloroflexi are often observed to have oligotrophic attributes^63–65^, and were thus widely distributed and able to sustain viable populations in the different habitats in this study (Fig. 3). However, whether this pattern is attributable to the differences in current environmental factors (e.g. physicochemical properties, nutrient availability, antagonistic factors, etc.) or historical exposures (e.g. availability for microbial colonization) is still unclear. Further well-organized experiments and quantitative analyses are required to identify the determinants that shape the microbial patterns among the distinct habitats in the grassland ecosystem.

### Keystone species play important roles in connecting different microorganisms among entire habitats

As previously mentioned, the microbial structures were distinct among the different habitats which is likely due to niche differentiation and the habitat preferences of species. Species selection may also be a consequence of the biological interactions between microbes, e.g. mutualistic, competitive, and syntrophic relationships. The relationships were visualized using a co-occurrence network, which revealed high connectivity between different microorganisms. The network structure was modularized and the nodes in the different modules indicate similar functions or share similar preferred environmental conditions among groups^66^. For example, some bacteria in module I are involved in plant litter degradation. Fungal genera *Mortierella*^67^ and *Acremonium*^68^, as well as bacterial genera *Roseiflexus*^69^ and *Rubrobacter*^70^ that contain various lignocelluloses, xylanases, and glycoside hydrolases, may play important roles in cellulose, hemicellulose, or lignin breakdown. The main taxa in module II may be involved in biogeochemical C- and N-cycles. *Nitrospira* participate in a nitrification process that is important in the biogeochemical nitrogen cycle. *Mesorhizobium* are common nitrogen-fixing symbiotic bacteria that also play significant roles in the nitrogen cycle^71^. Other bacteria in module II, such as Nocardioidaceae and Nitrosomonadaceae, are reportedly C- and N-cycling participants^72^. Microorganisms in module III are mainly associated with plant protection and growth. Members of Actinobacteria are known to serve as biocontrol agents against soil-borne plant pathogens via the production of different kinds of secondary metabolites and biologically active substances, such as enzymes and antibiotics^73^. In a previous study, genera belonging to Actinobacteria in module III, such as *Actinoplanes*, *Micromonospora*, and *Nocardioides*, were found to be involved in the resistance against plant disease^74^. Other bacteria, including *Sphingomonas*^75^, *Microvirga*^76^, *Methylobacterium*^77^, and *Bacillus*^78^ members, were also shown to be antagonistic to plant pathogens or beneficial in forming nitrogen-fixation symbioses to promote plant growth. The microorganisms of module III were obviously distributed with a bias for the phyllosphere habitat, indicating a shared habitat preference. The co-occurrence patterns of microbial communities in the three different habitats tended to be non-random and function-driven^79^.

By discerning the different roles of the nodes in the co-occurrence network, *Zi*-*Pi* plots have important implications for keystone species definition in microbial communities within a particular environment^80–82^. In the present study, eight OTUs were identified as the keystone taxa that play important roles in connecting the different microbiomes (Fig. 4B). *Microvirga* are reportedly beneficial microbes in the soil-plant ecosystem, i.e. by improving soil nutrients, promoting plant growth, and controlling soil-borne diseases^76,83^. In the present study, this genus was detected in all three habitats and identified as a keystone taxon in the network analysis, which indicates the significant role of its representatives in promoting the health of grassland ecosystems. *Nitrospira* was another keystone taxon detected in all three habitats, and which contains well-known nitrite oxidizers^84^. This genus may, therefore, play vital roles in the biogeochemical nitrogen cycle in the animal excrement-plant-soil system. *Blastococcus* were reported as surface active compounds-producing bacteria^85^, and potential bioemulsifiers produced by this genus may be useful for the biodegradation and bioremediation of pesticides or other hydrocarbon pollutants in grasslands^86^. *RB41* have been identified as sensitive biomarkers that respond to changes of fertilization conditions^87^, thus a series of corresponding responses may occur in response to variations in soil nutrient availability. Besides, members of norank bacteria were also identified as keystone taxa in this study, however, further analyses of their potential roles in ecological functioning and processes are still required.

In summary, this study presents the significant difference in microbial communities among three distinct habitats (animal excrement, phyllosphere, and soil) in the Xilingol steppe grassland. A large number of microorganisms were detected in the individual habitat types, of which only 7.81% of the microbial OTUs were shared among the three habitats. Our findings suggest a strong niche differentiation and habitat filtering of microbial communities in the grassland ecosystem. However, the coexisting microorganisms comprised over 60% of the sequences, despite their limited population, which indicates that they have a high dispersal potential. Moreover, eight microbial OTUs were identified as the keystone taxa in this study, which indicates their significant role in the complex ecological interactions across the different microorganisms in the grassland ecosystem. Overall, this is the first report that directly compares the microbial compositions of different habitats in a grassland. Further studies are needed to elucidate how this habitat filter affects the ecological processes and functioning of the grassland ecosystem.

## Materials and Methods

### Study sites and sample collection

This study was conducted in the Xilingol steppe grassland (44°01′N, 116°24′E), in the central-east of Inner Mongolia Autonomous Region, China. The mean annual precipitation and temperature of this region is 195 mm and 5.1 °C, respectively^88^. The sampling sites were at an elevation of 1100 m. The dominant soil type of the study site was chestnut loam and the dominant vegetation species were *Leymus chinensis*, *Agropyron cristatum*, and *Stipa grandis*. The main large herbivores that inhabit the Xilingol steppe include Ujimqin sheep, Luxi cattle, and Arabian horses. A sampling transect was created (50 m × 50 m) and three different vegetation types as well as three animal species were selected. Animal excrement samples and grass samples were collected in triplicate in each sampling area. Corresponding soil samples were collected from two different depths, comprising the shallow soil samples (10–20 cm below ground, three replicate plots) and deep soil samples (70–100 cm, three replicate plots), determined by a steel probe (1 m). Soil samples were sieved to 2 mm particle size for subsequent experiments. All samples were labelled and stored at –80 °C until use for DNA extraction.

### DNA extraction, PCR, and sequencing

Genomic DNA from the all samples was extracted using a MoBio PowerSoil kit (MoBio Laboratories, Carlsbad, CA, USA) according to the manufacturer’s instructions. Genomic DNA from the grass samples were extracted using a modified method of Zhao *et al*.^89^. Briefly, the phyllosphere microorganisms were detached from the grass surface by shaking the grass samples in sterile water, in a 1:10 ratio, at 25 °C; the treated grass samples were subjected to ultrasonic disruption for 3 min at 300 W; the same volume of sterile water was added to the grass samples and the ultrasonication was repeated four times. Microbial enrichment was then performed by passing the liquid through a 0.22 μm mixed cellulose ester membrane (Millipore, Massachusetts, USA). Genomic DNA from the phyllosphere microorganisms in the enriched membrane was extracted using the MoBio PowerSoil kit after membrane shearing. The final DNA samples obtained were a mixture of triplicate extracted DNA in the same volume from the three replicates in the same samples, and were stored at −80 °C until further analysis.

For the Illumina MiSeq sequencing, universal bacterial primers 338F (5′-ACTC CTACGGGAGGCAGCAG-3′) and 806R (5′-GGACTACHVGGGTWTCTAAT-3′), that target the V3–V4 region of the 16S rRNA gene, were used to create the bacterial libraries^90^. Primers 524F-10-ext (5′-TGYCAGCCGCCGCGGT AA-3′) and arch958R (5′-YCCGGCGTTGAVTCCAATT-3′), specific to the V4–V5 region of the archaeal 16S rRNA gene, were used to create the archaeal libraries^91^. Primers ITS1F (5′-CTTGGTCATTTAGAGGAAGTAA-3′) and ITS2R (5′-GCTGCGTTCTTCATCG ATGC-3′) were used to create fungal libraries^92^. Three replicates were conducted for each DNA sample and a negative control containing 1 μL sterile water was prepared to determine the PCR reagent contamination. PCR program conditions were as follows: initial denaturation at 95 °C for 3 min; 35 cycles of 95 °C for 30 s, 55 °C for 30 s, and 72 °C for 45 s; and a final extension at 72 °C for 10 min. Three independent PCR products for each DNA sample were then mixed to prepare the PCR amplicon library to minimize the impact of potential early-round PCR errors^93^. The PCR products were quantified and homogenized with a QuantiFluor^TM^-ST (Promega, Madison, WI, USA) and were sent for sequencing on the Illumina MiSeq PE300 platform by the Genomic Research Center at Majorbio Bio-pharm Technology Co., Ltd. (Shanghai, China).

### Sequence data analysis

Before analysis, raw sequences with a quality score below 30 and containing ambiguous base (“N”) were trimmed by using Trimmomatic^94^. Barcode and primer sequences, as well as sequences shorter than 200 bp or longer than 550 bp were also removed. Pair-end sequences were merged into full-length sequences using FLASH software^95^. Chimeras were identified and discarded using the “chimera.uchime” command in Mothur package (v 1.30.1)^96^. Similar sequences were clustered into OTUs with a 97% similarity using USEARCH v7.0^97^. For bacterial and archaeal libraries, the Silva database (release 128) was used for taxonomic assignment of OTUs using RDP classifier (v2.2)^98^. For fungal libraries, OTUs were classified with reference to the UNITE database (release 6.0). Non-target sequences, including mitochondria, chloroplasts, and non-fungal eukaryotic sequences were also removed from the final OTU dataset by QIIME^99^.

### Statistical analysis

Alpha diversity indices, including richness, Chao 1 estimator, Shannon index, and Good’s coverage of samples were calculated using Mothur^96^. The compositions of three microbial groups, i.e. bacteria, archaea, and fungi, were displayed using an average relative abundance for each taxa with R software^100^. To better convey the biological information in these samples, the bacterial communities were visualized at the phylum level, whereas the archaeal and fungal communities were displayed at the class level. The PCoA, based on the Bray Curtis distance matrix, was used to assess the beta diversity patterns to determine the dissimilarity of the microbial community compositions among the three habitats: animal excrement, phyllosphere, and soil. To determine the differences in the microbial community compositions of the different habitats, we used a permutational multivariate analysis of variance (PERMANOVA) in R software. The LEfSe, which uses a non-parametric factorial Kruskal-Wallis (KW) sum-rank test and performs a linear discriminant analysis (LDA) to evaluate the effect size of each taxa^101^, was performed using an online LEfSe program (http://huttenhower.sph.harvard.edu/galaxy/root?tool_id=lefse_upload) to identify the microbes that differed significantly in abundance among the three habitats.

A network analysis was performed using Molecular Ecological Network Analysis Pipeline^102–104^, to gain a comprehensive understanding of the microbial interactions among the different microbiomes. The OTUs with more than five sequences were used because they are more representative in the network analyses^105^. The OTUs that occurred in more than half of all samples were kept in the pipeline using default settings. A similarity matrix was constructed based on Pearson’s Correlation Coefficient between pairwise OTUs. A cutoff value (similarity threshold, *St*, for network construction) was chosen according to the P value of the similarity matrix (P > 0.05). After filtering the pairwise OTUs, i.e. those with abundances lower than the *St*, a new OTU table was generated for the subsequent network construction. After the “global network properties”, “individual nodes’ centrality”, and “module separation and modularity calculation” were calculated, the networks were visualized with the interactive platform Gephi^106^.

## Supplementary information

Supplementary materials

## Data Availability

The sequence data obtained in this study have been deposited in the NCBI short read archive (SRA) under the Bioproject accession number PRJNA392724, with Biosample numbers SAMN07305413–SAMN07305415.
