# Supplementary material for: Habitat filtering shapes the differential structure of microbial communities in the Xilingol grassland
Source: Sci Rep. 2019 Dec 18;9:19326. doi: 10.1038/s41598-019-55940-y (PMC6920139; doi:10.1038/s41598-019-55940-y)
Supplement: Supplementary file 1 — Supplementary materials [file 41598_2019_55940_MOESM1_ESM.pdf]

Scientific Reports

Habitat filtering shapes the differential structure of microbial communities in the Xilingol grassland

Jie Yang[1], Yanfen Wang[2], Xiaoyong Cui[2], Kai Xue[1], Yiming Zhang[3], Zhisheng Yu [1,4]*

[1]*College of Resources and Environment, University of Chinese Academy of Sciences, Beijing 100049, China;* [2]*College of Life Sciences, University of Chinese Academy of Sciences, Beijing 100049, China;* [3]*Beijing Municipal Ecological Environment Bureau, Beijing 100048, China;* and [4]*Research Center for Eco-Environmental Sciences, Chinese Academy of Sciences, Beijing 100085, China*

*All correspondence should be addressed to:

Prof Zhisheng Yu

College of Resources and Environment

University of Chinese Academy of Sciences

19 A Yuquan Road, Shijingshan District

Beijing 100049, P. R. China

E-mail: yuzs@ucas.ac.cn

Tel: +86 10 88256057

Fax: +86 10 88256057

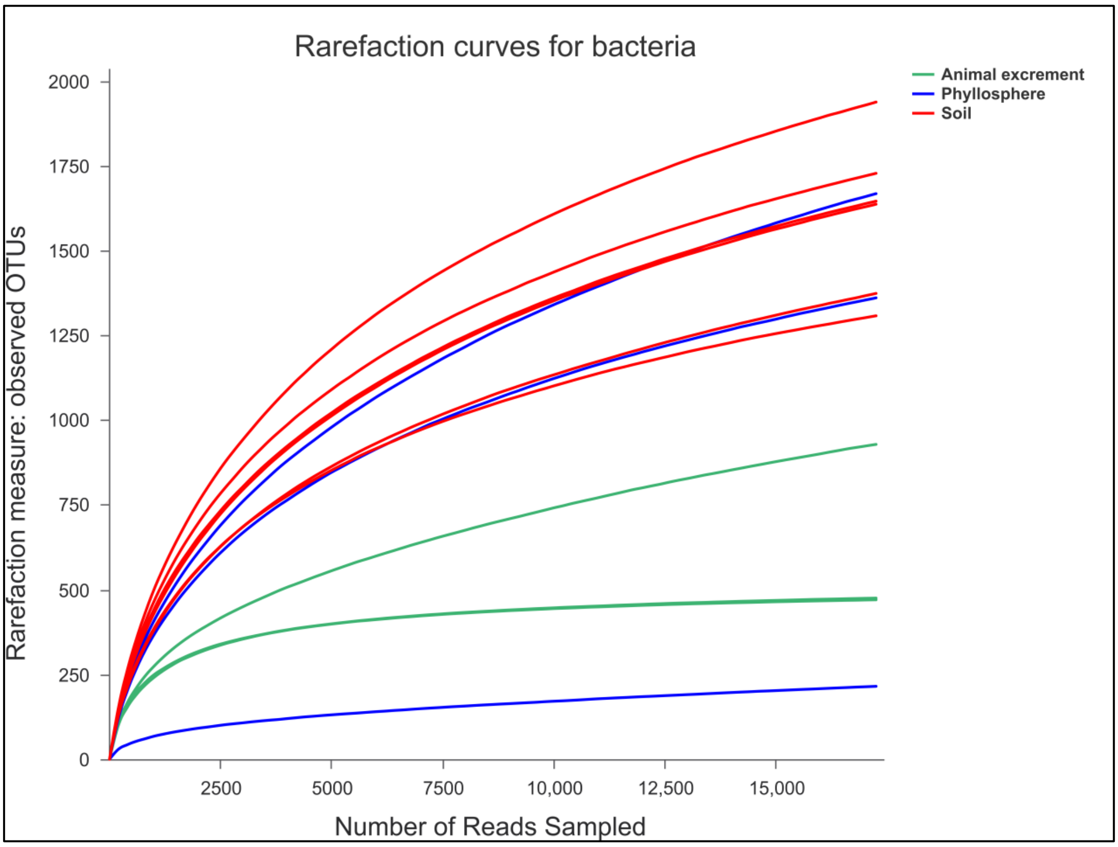

Supplementary Fig. S1 Rarefaction curve for bacteria

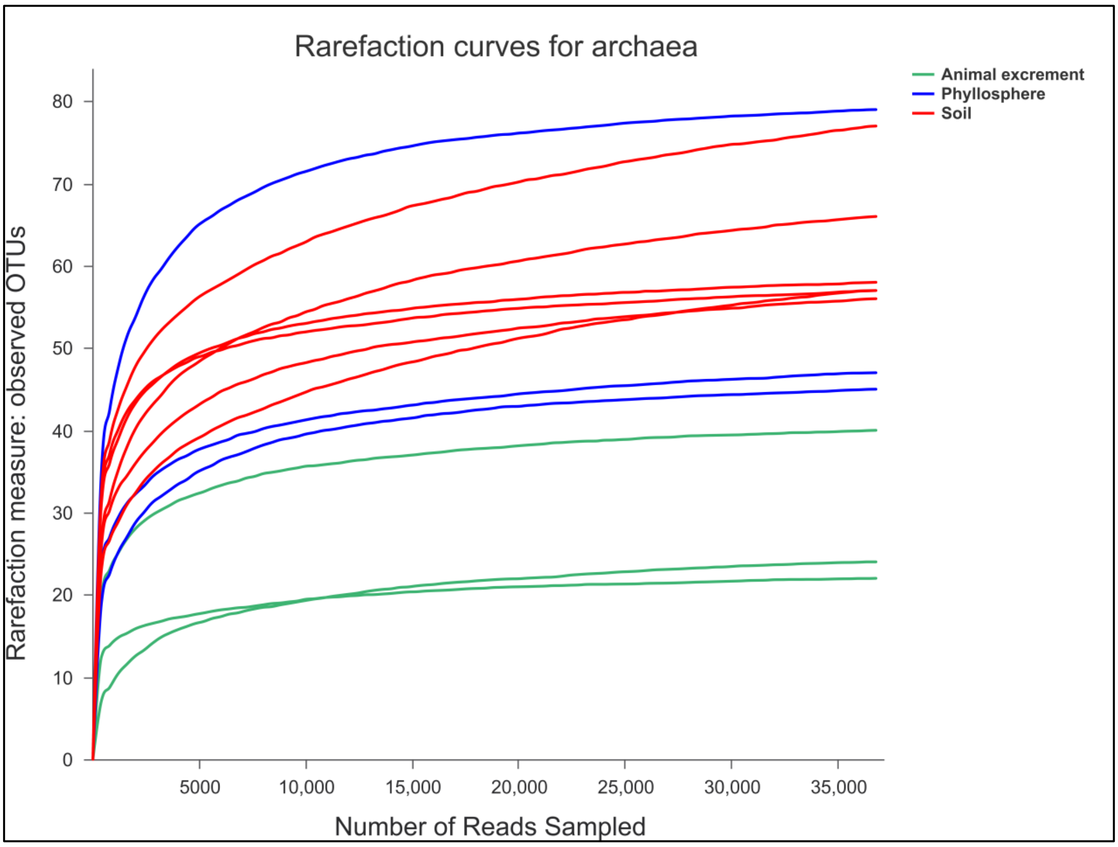

Supplementary Fig. S2 Rarefaction curve for archaea

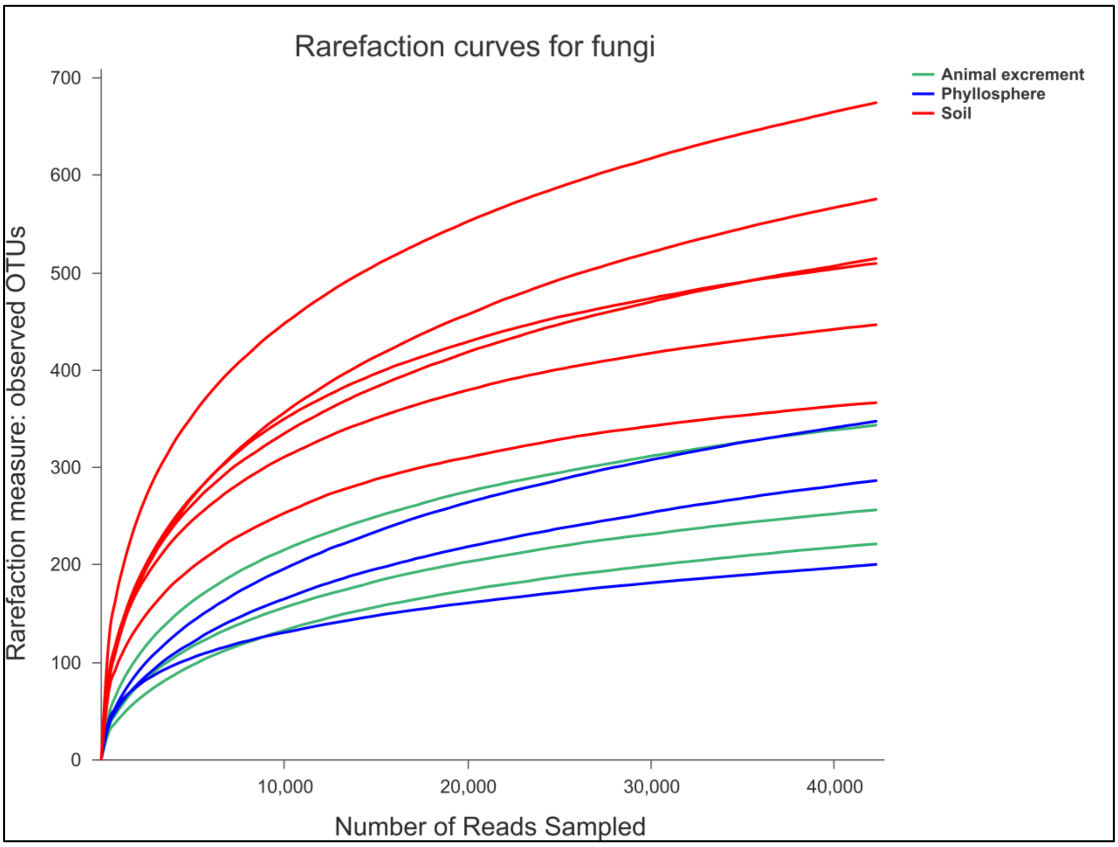

Supplementary Fig. S3 Rarefaction curve for fungi

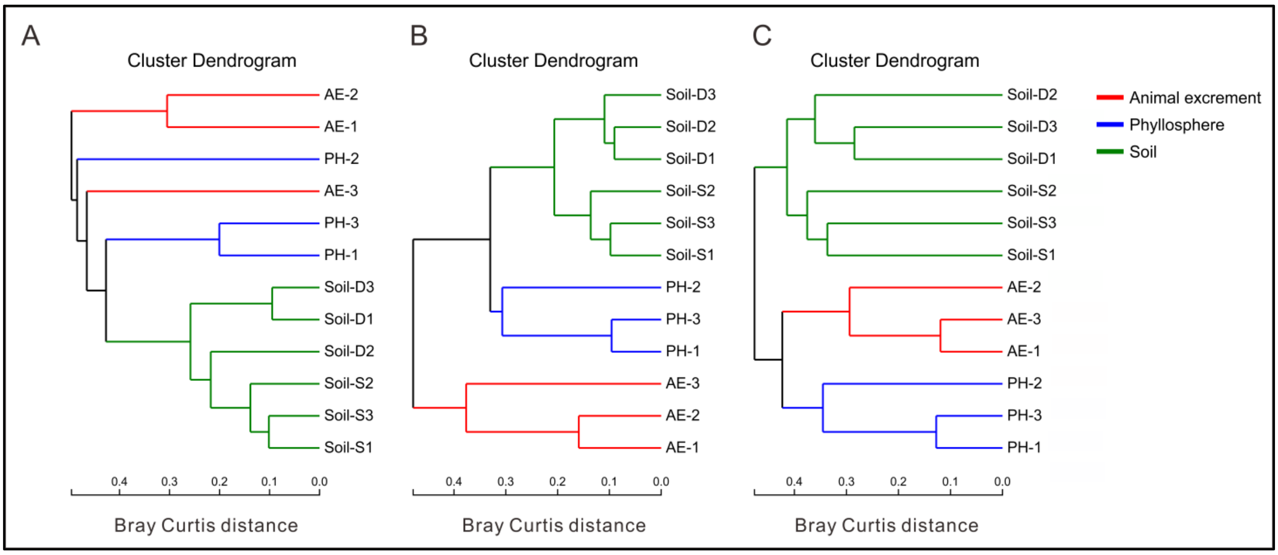

Supplementary Fig. S4 Hierarchical cluster dendrogram of different samples for bacteria, archaea, and fungi (A–C) based on a Bray-Curtis distance matrix. AE, animal excrement; PH, phyllosphere; Soil-S, shallow soil; Soil-D, deep soil

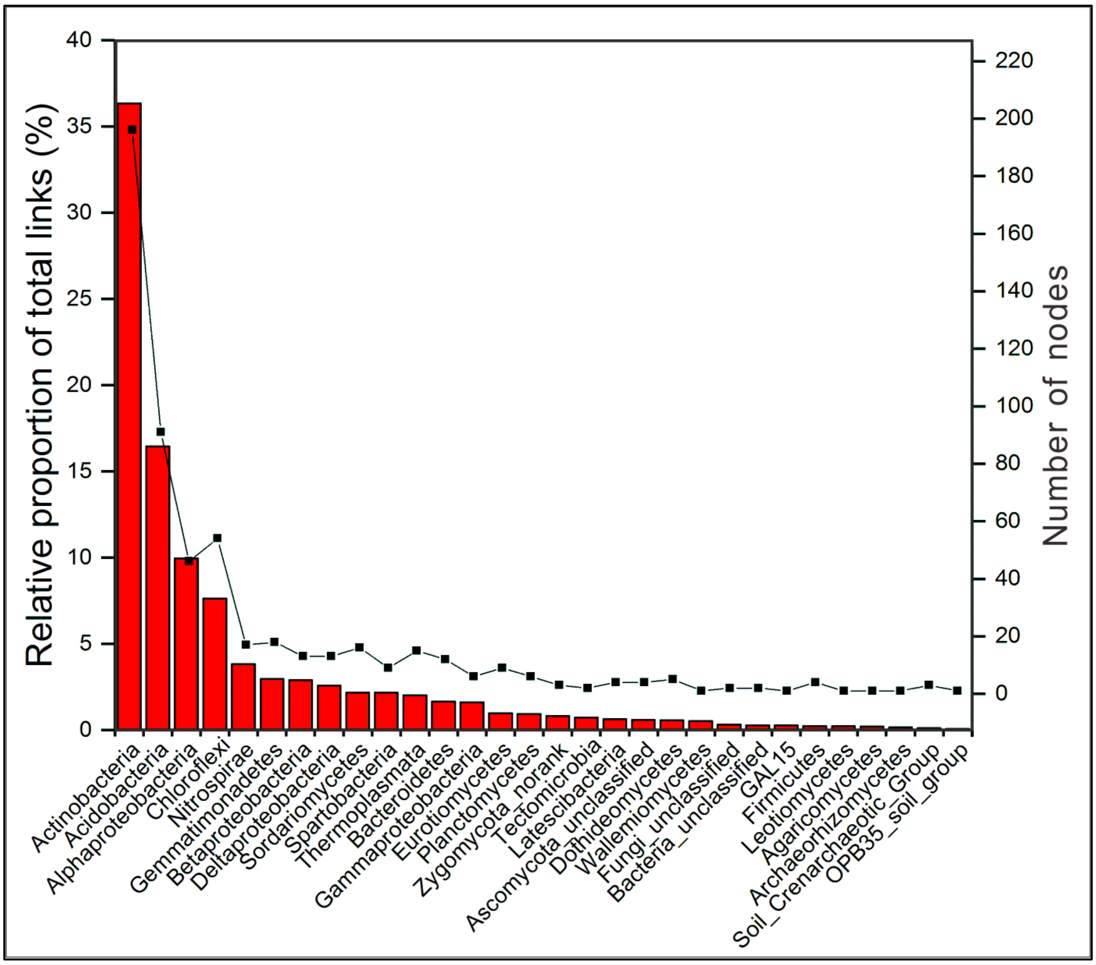

Supplementary Fig. S5 Distribution of network nodes in different microbial phyla or classes. The levels of classification are the same as those used in Fig. 1

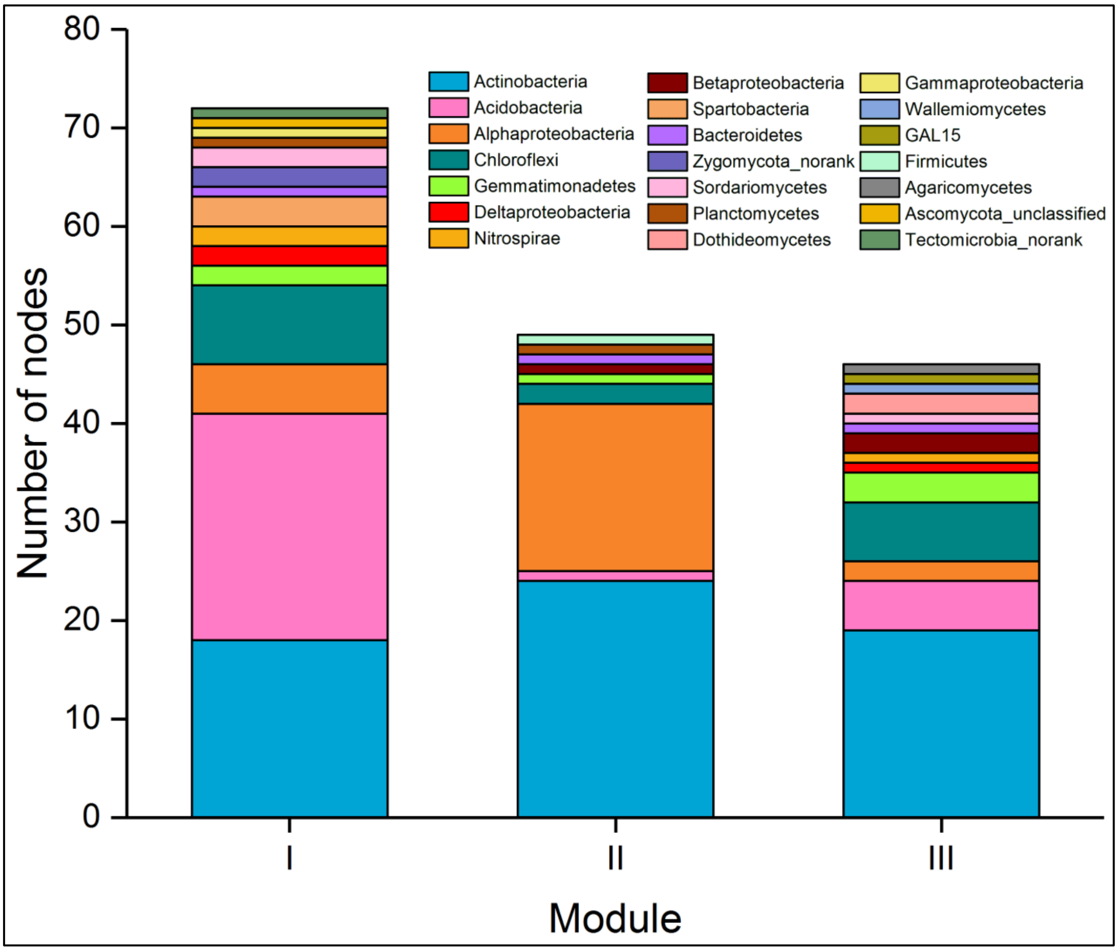

Supplementary Fig. S6 Taxonomic distribution of three major modules in the co-occurrence network. The levels of classification in the different modules are the same as those used in Fig. 1

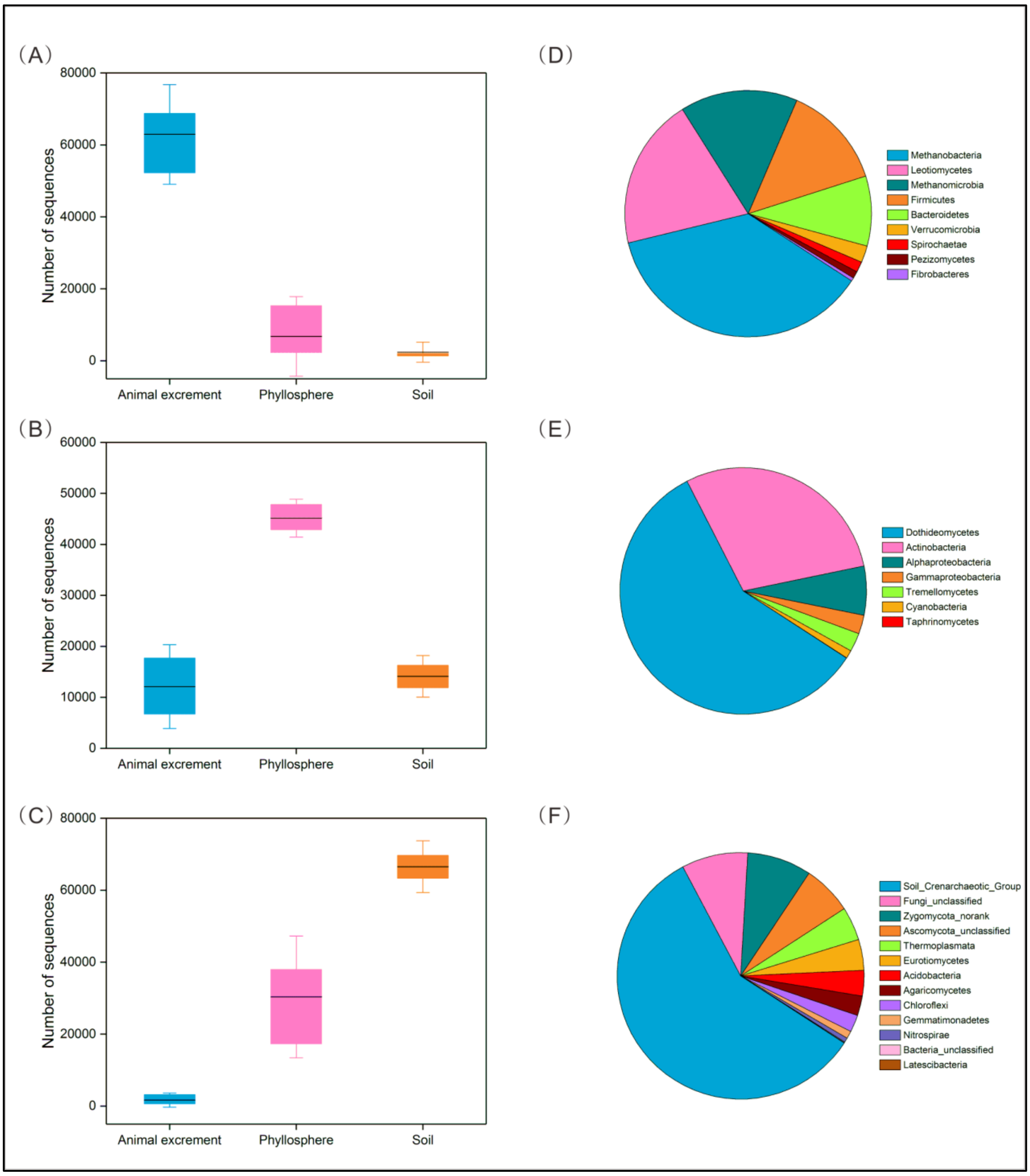

Supplementary Fig. S7 Taxonomic composition and abundance patterns of microorganisms in three different grassland habitats. Dominant microorganisms in animal excrement, the phyllosphere, and soil are presented in A–C, respectively. Corresponding taxonomic data are presented as contributions of each microbial phylum (bacteria) or class (archaea and fungi) to the total sequences (D–F)

Supplementary Table S1 Comparisons of microbial dissimilarity among the different grassland habitats: animal excrement, phyllosphere, and soil

Microbial group	Pair of samples compared	Bray Curtis index	Number of shared OTUs	Percentage of shared OTUs	Percentage of sequences in shared OTUs
Total	Animal excrement and Phyllosphere and Soil	0.906	493	7.81	61.13
	Animal excrement and Phyllosphere	0.908	681	17.72	74.59
	Animal excrement and Soil	0.969	859	16.10	62.93
	Phyllosphere and Soil	0.841	1524	27.59	75.40
Bacteria	Animal excrement and Phyllosphere and Soil	0.946	336	7.67	33.06
	Animal excrement and Phyllosphere	0.988	399	13.24	21.42
	Animal excrement and Soil	0.973	618	17.21	49.32
	Phyllosphere and Soil	0.899	1221	32.78	71.54
Archaea	Animal excrement and Phyllosphere and Soil	0.839	25	15.24	81.97
	Animal excrement and Phyllosphere	0.889	35	30.70	82.54
	Animal excrement and Soil	0.992	27	23.68	81.80
	Phyllosphere and Soil	0.660	41	26.80	92.57
Fungi	Animal excrement and Phyllosphere and Soil	0.932	132	7.47	54.46
	Animal excrement and Phyllosphere	0.846	247	34.55	89.38
	Animal excrement and Soil	0.944	214	13.12	52.07
	Phyllosphere and Soil	0.963	262	15.93	62.03
